# Roles of Ferredoxin-NADP^+^ Oxidoreductase and Flavodoxin in NAD(P)H-Dependent Electron Transfer Systems

**DOI:** 10.3390/antiox11112143

**Published:** 2022-10-29

**Authors:** Takashi Iyanagi

**Affiliations:** Graduate School of Life Science, University of Hyogo, 3-2-1 Koto, Akoh 678-1297, Hyogo, Japan; takashi.iyanagi@gmail.com

**Keywords:** ferredoxin-NADP^+^ oxidoreductase (FNR), ferredoxin (Fd), flavodoxin (Fld), diflavin reductase family, catalytic cycle, electron transfer, redox potentials, evolutionary aspects

## Abstract

Distinct isoforms of FAD-containing ferredoxin-NADP^+^ oxidoreductase (FNR) and ferredoxin (Fd) are involved in photosynthetic and non-photosynthetic electron transfer systems. The FNR (FAD)-Fd [2Fe-2S] redox pair complex switches between one- and two-electron transfer reactions in steps involving FAD semiquinone intermediates. In cyanobacteria and some algae, one-electron carrier Fd serves as a substitute for low-potential FMN-containing flavodoxin (Fld) during growth under low-iron conditions. This complex evolves into the covalent FNR (FAD)-Fld (FMN) pair, which participates in a wide variety of NAD(P)H-dependent metabolic pathways as an electron donor, including bacterial sulfite reductase, cytochrome P450 BM3, plant or mammalian cytochrome P450 reductase and nitric oxide synthase isoforms. These electron transfer systems share the conserved Ser-Glu/Asp pair in the active site of the FAD module. In addition to physiological electron acceptors, the NAD(P)H-dependent diflavin reductase family catalyzes a one-electron reduction of artificial electron acceptors such as quinone-containing anticancer drugs. Conversely, NAD(P)H: quinone oxidoreductase (NQO1), which shares a Fld-like active site, functions as a typical two-electron transfer antioxidant enzyme, and the NQO1 and UDP-glucuronosyltransfease/sulfotransferase pairs function as an antioxidant detoxification system. In this review, the roles of the plant FNR-Fd and FNR-Fld complex pairs were compared to those of the diflavin reductase (FAD-FMN) family. In the final section, evolutionary aspects of NAD(P)H-dependent multi-domain electron transfer systems are discussed.

## 1. Introduction

Land plant and cyanobacterium ferredoxin (flavodoxin)-NADP^+^ oxidoreductases (FNRs) catalyze the reversible electron transfer that occurs in the photosynthetic reaction (formation of NADPH) and non-photosynthetic reactions (NADPH-dependent redox metabolic pathways) (Equation (1)) [1,2,3,4,5,6]. The forward and reverse reactions of Equation (1) are catalyzed by distinct FNR and ferredoxin (Fd) isoforms [7]:

2Fd (Fe^2+^) + NADP^+^ + H^+^ ⇌ 2Fd (Fe^3+^) + NADPH (1)

In cyanobacteria and some algae, FMN-containing flavodoxin (Fld) acts as a one-electron carrier instead of ferredoxin (Fd) under iron-limiting conditions [8]. Meanwhile, the FNR- and Fld-like domains are present in eukaryotic NAD(P)H-dependent enzymes, including cytochrome P450 reductase (cyt P450 reductase) [9,10] and nitric oxide synthase (NOS) isoforms [11]. The catalytic domains of these enzymes share a high sequence similarity and structure with FNR and Fld, where the FAD-FMN redox pair donates electrons to final electron acceptors during sequential one-electron transfer reactions [12]. NADH-cytochrome *b*_5_ reductase (cyt *b*_5_ reductase) shares structural similarities with plant FNR, despite its amino acid sequence similarities being lower [13]. The cyt *b*_5_ reductase-cyt *b*_5_ complex pair donates an electron to metal-containing proteins [14]. Cyt P450 reductase (FAD-FMN) and the cyt *b*_5_ reductase (FAD)-cyt *b*_5_ complex exhibit diverse functions in regard to the electron acceptors.

In addition to plant FNR, cyt P450 reductase and NOS isoforms catalyze a one-electron reduction of artificial electron acceptors such as quinone derivatives, where the resulting quinone radical reacts with molecular oxygen to form the superoxide radical [15,16,17,18,19], while NAD(P)H: quinone oxidoreductase (NQO1) bears a common flavodoxin-like topology in its active site [20] and catalyzes the two-electron reduction of anticancer quinone derivatives; the resulting hydroquinone form is primarily conjugated by UDP-glucuronosyltransferase (UGT) and sulfotransferase (SULT) [21]. Thus, the NQO1-UGT/SULT pair systems function as potent antioxidant enzyme systems. In this review, the biochemical and structural aspects of plant FNR, Fd and Fld are briefly described, and the catalytic cycles of the FNR-Fd and FNR-Fld complex pairs are compared with those of the diflavin reductase family. In the final section, evolutionary events of electron transfer systems including FNR, Fd and Fld modules are discussed.

## 2. Structure and Properties of the FNR, Fd and Fld

The three-dimensional structure of FNR from spinach reveals two subdomains, the NADPH-binding domain and the FAD-binding domain [1,5]. FNR can transfer electrons to both Fd [2Fe-2S] and Fld (FMN), where an interaction with Fd or Fld occurs in the same structural region of the FAD-binding domain [1,22]. Both Fd and Fld act as one-electron acceptors or donors, and Fd is replaced with Fld under low-iron conditions [8,23]. The rate of electron transfer among these proteins is controlled by several factors, including the relative orientation and distance between protein–protein interactions and the differences in the redox potentials between the protein-bound donor and acceptor redox cofactors [1,3,23,24,25,26]. Sinohara et al. [1] reported the crystal structures of the *R*FNR-*R*Fd (Fd III) (*R* for root) and *L*FNR-*L*Fd (Fd I) (*L* for leaf) complexes, and this provides a structural basis for reversing the redox pathway. In the *L*FNR-Fd complexes, the distance between the [2Fe-2S] cluster of Fd and the dimethylbenzene edge of the FAD ring is ~5.5–6.0 Å [1,24], while the *R*FNR-Fd complexes can utilize the different sides of the [2Fe-2S] cluster for intermolecular electron transfer. The modeling of the FNR-Fld complexes indicates that the distance between the two flavin rings is ~4.1 Å and that no intervening residues are present between the two cofactors, thus making possible direct one-electron transfer [25]. Taken together, these observations suggest that the complexes between oxidized FNR and oxidized Fd are relatively stable, as the complexes are stabilized by a salt bridge of FNR33Lys-Fd60Asp between FNR and Fd [26]. However, this stability is decreased by NADP(H) binding, and unstable complexes can promote the electron transfer rate during the catalytic cycle [27]. FNR interacts with Fd or Fld in photosynthetic and non-photosynthetic reactions.

The isoalloxazine ring of FAD in the leaf and root FNR is sandwiched between two aromatic residues (Tyr314 and Tyr95), where the phenol ring of carboxyl(C)-terminal Tyr314 shields the face of the isoalloxazine ring of FAD [5,28]. Thus, C-terminal Tyr314 must move away for productive hydride transfer from reduced FAD to NADP^+^, and Ser96 interacts with the N5 atom of the isoalloxazine ring of FAD, where Ser96 forms a hydrogen bond with Glu312. Additionally, C-terminal Tyr314 and the Ser96-Glu312 pair modulates the affinity for NADP^+^, the stabilization of FAD semiquinone and the rate of electron transfer [29]. Therefore, Tyr314 and the Ser96-Glu312 pair are involved in the modulating of the flavin redox properties [30,31].

The formation of the reduced FAD-NADP^+^ (FADH^−^-NADP^+^) complex with the charge transfer bands of 500–750 nm proceeds via an FAD_ox_-NADPH charge-transfer species [32]. In the presence of excess NADPH (>10-fold), bound NADP^+^ is replaced by NADPH: FADH^−^-NADP^+^ + NADPH ⇌ FADH^−^-NADPH + NADP^+^, where the FADH^−^- NADPH complex does not exhibit significant charge transfer bands. This could be caused by a decrease in π−π stacking interactions between reduced FAD and NADPH. The value of *E*_ox/red_ measured using dithionite as a reductant is −377 mV [32], which is fitted to a Nernstian *n* = 2 curve. However, the two-electron reduction potential in the presence of NADP^+^ is divided into *E*_ox/sq_ (−306 mV) and *E*_sq/red_ (−386 mV) (Table 1), suggesting that the redox potentials of FNR are regulated by the NADP^+^/NADPH ratio.

**Scheme 1:**
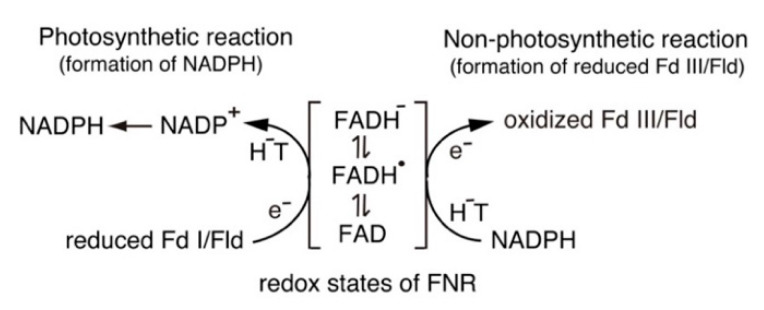
Switching between one-electron and two-electron transfer reactions in the FNR-Fd I/Fld and FNR-Fd III/Fld systems. H^−^T, hydride transfer.

The redox potentials for *L*Fd I and *R*Fd III are −401 mV and −321 mV, respectively. [33]. Thus, the one- and two-electron redox potentials of FNR are important factors that control the electron transfer rate (Scheme 1). For the spinach *L*FNR, the *E*_m_ (FAD/FADH^−^) value is −343 mV. Regarding the one-electron redox potential, the oxidized-semiquinone couple (*E*_ox/sq_) is −350 mV, and the semiquinone-fully reduced couple (*E*_sq/red_) is −335 mV at pH 7.0 [34] (Table 1). Thus, the electron transfer from Fd I to NADP^+^ is thermodynamically favorable. On the other hand, the electron transfer from NADPH to Fd III is relatively favorable. In both systems, tissue-specific FNR mediates reversible electron transfer reactions (Equation (1) and Scheme 1) [33].

Fld is a small electron carrier that participates in low-redox-potential electron transfer pathways, which are classified into three groups based on the presence of tryptophan (Trp) and tyrosine (Tyr) near the isoalloxazine ring of FMN [35]. In all Flds, the semiquinone formation constant *K*s value is larger than unity, indicating that the *E*_ox/sq_ value is always more positive than the *E*_sq/red_ value [36]. The *E*_ox/sq_ value is associated with the proton-coupled one-electron reduction from FMN to FMNH^•^, while *E*_sq/red_ is associated with the one-electron reduction from FMNH^•^ to FMNH^−^. The *E*_ox/sq_ value is shifted from ~−240 mV to less-than-negative values, while *E*_sq/red_ is shifted from ~−170 mV to ~−540 mV, depending on the pH and the species of Fld. The neutral semiquinone, FMNH^•^, is stabilized by the hydrogen bond of the carbonyl and amide groups of the protein backbone with the N5 atom of the isoalloxazine ring of the flavins. Its reactivity is lower than that of the fully reduced form, where the FMNH^•^/FMNH^−^ couple acts as a one-electron carrier [36].

Flds possess a unique structure, and the loop structures in the FMN environment play an important role in electron transfer reactions [37]. The FMN binding sites in the bacterial Fld and eukaryotic diflavin reductase family possess similar loop structures and are sandwiched between two aromatic amino acid residues. Rwere et al. [38] reported that the length and sequence of the “140 s” FMN binding loop of cyt P450 reductase functions as a key determinant of its redox potential and activity with cyt P450s. The one-electron redox potentials of cyt P450 reductase (FAD-FMN) [39,40] and the reductase domain (FAD-FMN) of NOS isoforms [41,42] are different from those of plant FNR (FAD) [34] and *Anabaena* flavodoxin (FMN) [43], but the one-electron redox potentials of FAD and FMN always satisfy *E*_ox/sq_ > *E*_sq/red_ (Ref. [14] and Table 1).

The kinetic parameters for Fd reduction by FNR using the NADPH-dependent cyt *c* reductase assay (NADPH → FNR → Fd/Fld → cyt *c*) were reported [29]. The values for pea FNR are in the range of those of the *Anabaena* enzyme, with a slightly larger *k*_cat_ and a smaller *K*_m_ (Table 2) [29], while Kimata-Ariga and co-workers recently reported the tissue-specific parameters, where maize *R*FNR has a higher *K*_m_ value for Fd I than for Fd III [1]. In addition, the orientation of the *R*FNR-Fd complex remarkably varies from that of the *L*FNR-Fd complex [1], suggesting that the root and leaf complexes utilize the different sides of the [2Fe-2S] cluster for the intermolecular electron transfer, which might lead to the evolutional switch between photosynthetic and heterotrophic assimilation.

## 3. Catalytic Cycle of the FNR-Fd and FNR-Fld Systems

### 3.1. Catalytic Cycle of the FNR-Fd System

**Figure 1:**
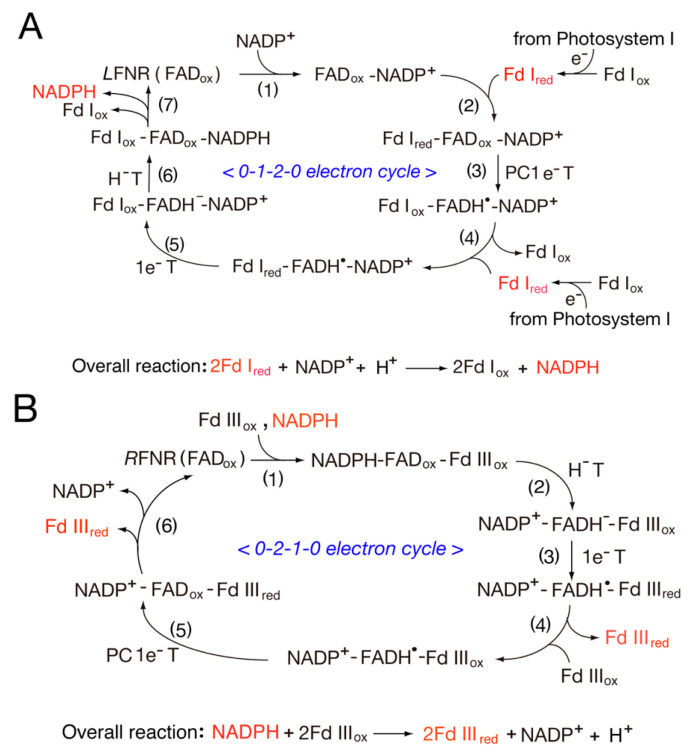
Proposed catalytic cycles of the *L*FNR (**A**) and *R*FNR (**B**) systems. (**A**) Photosystem I → *L*Fd I (2S-2Fe) → *L*FNR (FAD) → NADP^+^ [45] and (**B**) NADPH→*R*FNR (FAD) → *R*Fd III→metabolic pathways. H^−^T, hydride transfer; 1e^−^T, one-electron transfer; PC1e^−^T, proton-coupled one-electron transfer.

As already mentioned, the distinct isoforms of FNR and Fd are expressed in the different tissues, where *L*FNR and *L*Fd I are expressed in photosynthetic tissue, while *R*FNR and *R*Fd III are expressed in non-photosynthetic tissues. FNR mediates a switching between one-electron and two-electron transfer reactions (Scheme 1). Thus, the tissue-specific expression of the FNR and Fd isoforms indicates that different FNR and Fd isoforms participate in the different catalytic electron transfer processes (see Figure 1A,B). *L*FNR catalyzes the reduction of NADP^+^ to NADPH during the photosynthetic process in plants and cyanobacteria, where the two electrons from photosystem I are transferred from FADH^−^ to NADP^+^ in a process defined by: photosystem I (PSI) → Fd I [2Fe-2S] (−401 mV) → *L*FNR (FAD) → NADP^+^ (−320 mV). In non-photosynthetic reactions, NADPH generated by the photosynthetic reaction and the oxidative pentose phosphate cycle donates electrons to *R*Fd: NADPH (−320 mV) → *R*FNR (FAD) → Fd III (−321 mV) [33]. Thus, the photosynthetic process is more favorable than non-photosynthetic reactions. Both mechanisms are controlled by several factors: the redox potentials, the association/dissociation of complexes, and the distance between Fd [2Fe-2S] and FNR (FAD) in protein–protein interactions.

In photosystem I, the electron transfer sequence of the physiological reaction center is {P700* → A_o_ → A_1_ → FeS-X → [FeS-A → FeS-B]} → Fd I, in which Fd I accepts an electron from the [FeS-B] site (−580 mV), and the resulting reduced Fd I (−401 mV) binds to the oxidized FNR (FAD_ox_) [44], as shown in Figure 1A. Thus, Fd I functions as a mobile electron carrier. On the other hand, non-photosynthetic reactions begin from NADPH binding to the FAD_ox_-Fd III_ox_ complex: NADPH → *R*FNR (FAD)-*R*Fd III, as shown in Figure 1B.

In 1984, Batie and Kamin [45] proposed the catalytic cycle of the *L*FNR and *L*Fd I system (Figure 1A). This cycle involves basic electron transfer reactions, including one-electron transfer (1e^−^T), proton-coupled one-electron transfer (PC1e^−^T) and two-electron hydride transfer (H^−^T) reactions. The catalytic cycle includes steps 1–7, where the catalytic cycle begins from *L*FNR (FAD_ox_) (step 1). The oxidized Fd I accepts an electron from Photosystem I, and the resulting Fd I_red_ binds to the *L*FNR (FAD_ox_)-NADP^+^ complex (step 2). One electron is then transferred from Fd I_red_ to *L*FNR (FAD_ox_). In step 3, the one-electron transfer from reduced Fd I to oxidized FAD is coupled with proton transfer (PC1e^−^T). Electron transfer in this step is significantly decreased in D_2_O solution. In step 4, the Fd I_ox_ of the *L*FNR (FADH^•^)-NADP^+^ complex is replaced by reduced Fd I_red_, thus resulting in the Fd I_red_-*L*FNR (FADH^•^)-NADP^+^ complex. In step 5, the Fd_ox_-*L*FNR(FADH^−^)-NADP^+^ complex is formed via a one-electron transfer reaction (1e^−^T) from Fd I_red_ to *L*FNR (FADH^•^). In step 6, NADP^+^ accepts a hydride from FADH^−^ to form NADPH. In the final step, both Fd I_ox_ and NADPH are released; a new cycle then begins. This catalytic cycle is also supported by kinetic studies [46].

In 2003, Carrillo and Ceccarelli [47] proposed the “open questions” in regard to the Batie-Kamin catalytic model [45] on the basis of kinetics and binding experiments on spinach FNR. Currently, new data in regard to these open questions can be found (see Refs [1,48,49,50]).

In non-photosynthetic reactions (metabolic redox pathways) (Figure 1B), the reaction begins with *R*FNR (FAD_ox_). In the first step, NADPH binds to the FAD_ox_-Fd III_ox_ complex. In step 2, the complex NADP^+^-FADH^−^-Fd III_ox_ is formed via the hydride transfer (H^−^T) reaction, and this complex is followed by the formation of the NADP^+^-FNR (FADH^•^)-Fd III_red_ complex via one-electron transfer (1e^−^T) from FADH^−^ to Fd III_ox_ (step 3). In step 4, Fd III_red_ is released from the complex, and Fd III_ox_ binds. In step 5, the NADP^+^-FNR (FAD_ox_)-Fd III_red_ complex is formed via PC1e^−^T. In the final step, Fd III_red_ and NADP^+^ are released. Thus, reduced Fd III is formed in steps 3 and 5.

### 3.2. Catalytic Cycle of the FNR-Fld Systems

**Figure 2:**
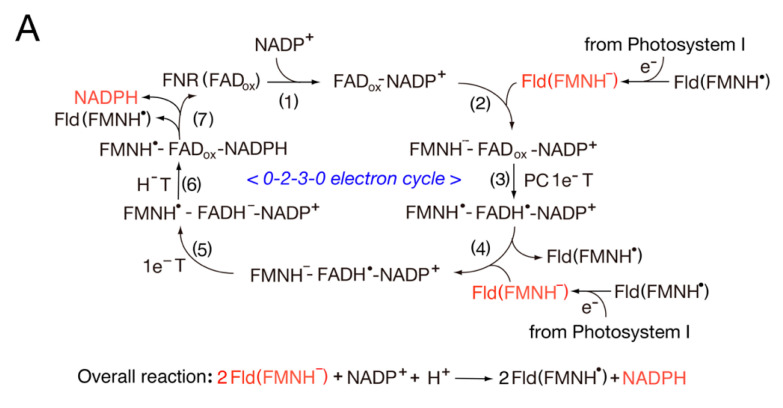

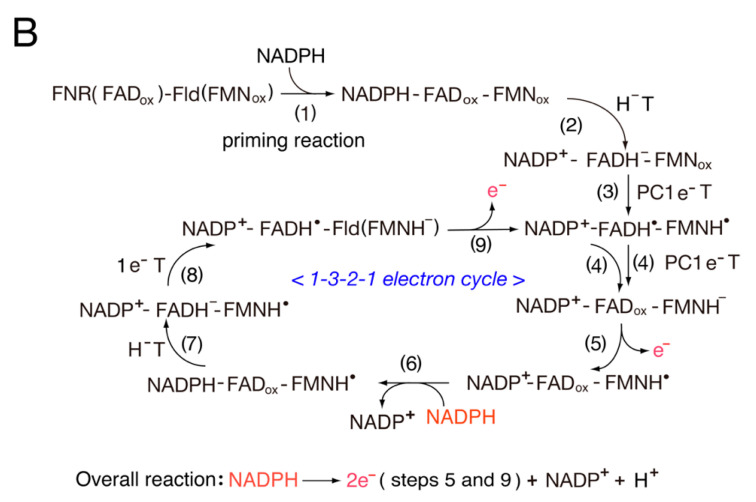
Proposed catalytic cycle of the FNR-Fld systems. (**A**) Photosystem I → Fld (FMN) → FNR (FAD) → NADP^+^ and (**B**) NADPH → FNR (FAD) → Fld (FMN) → metabolic redox pathways. In (**B**), the overall reaction is based on the catalytic cycle.

Fd can be replaced with FMN-containing Fld, where the FNR-Fd pair is replaced with the FNR-Fld pair in the catalytic cycle (Figure 2A). The FMN semiquinone (FMNH^•^) is highly stable. Thus, Fld functions as a one-electron carrier (FMNH^−^ ⇌ FMNH^•^ + e^−^). In the photosynthetic process, the catalytic cycle (Figure 2A) is similar to that presented in Figure 1A.

In the first step, NADP^+^ binds to *L*FNR (FAD_ox_). In step 2, reduced Fld (FMNH^−^) binds to FNR (FAD_ox_)-NADP^+^, and in step 3, the diflavin radical intermediate FMNH^•^-FADH^•^-NADP^+^ is formed via a PC1e^−^T reaction. Such intermediates are observed in the *Anabaena* PSI/Fld system [51]. In step 4, Fld (FMNH^•^) releases, and Fld (FMNH^−^) binds. In step 5, FMNH^•^-FADH^−^-NADP^+^ is formed during a 1e^−^T reaction, and in step 6, FMNH^•^-FAD_ox_-NADPH is formed during an H^−^T reaction. In the final step, Fld (FMNH^•^) and NADPH are released.

**Figure 3:**
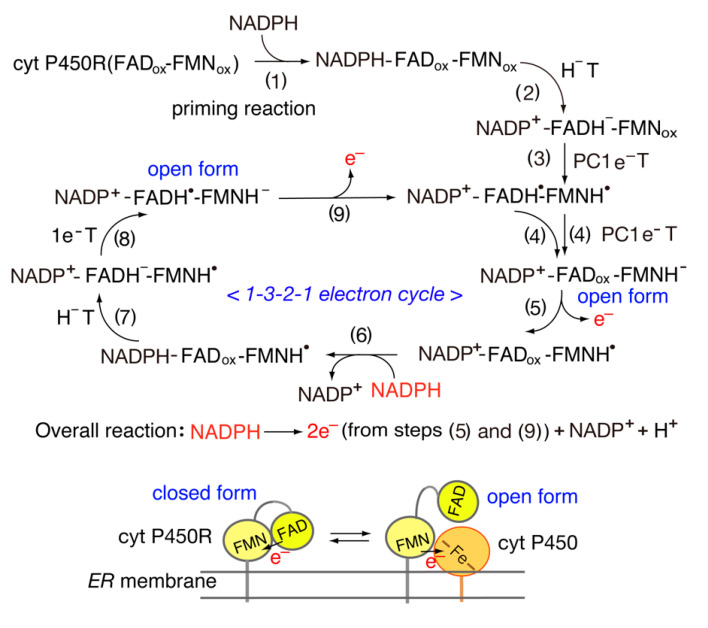
Proposed catalytic cycle of the eukaryotic-membrane-bound cyt P450 reductase-cyt P450 system. In this figure, the overall reaction is based on the catalytic cycle. Note that cyt P450R indicates cyt P450 reductase. H^−^T, hydride transfer; 1e^−^T, one-electron transfer; PC1e^−^ T, proton-coupled one-electron transfer.

The catalytic cycle of the non-photosystem includes the priming reaction (Figure 2B). The catalytic cycle begins from the complex NADP^+^-FAD_ox_-FMNH^•^ and includes the following sequences: (6) → (7) → (8) → (9) → (4) → (5), where the catalytic cycle shuttles among the 1-3-2-1 electron reduced states. The two electrons from steps 5 and 9 are transferred to the metabolic redox pathways. This overall catalytic cycle is very similar to that of cyt P450 reductase (see Figure 3), where the diflavin radical intermediate, FADH^•^-FMNH^•^, is formed through the catalytic cycle (step 9).

Recently, Utschig et al. [52] proposed a new approach for the electron transfer from photosystem I to Fd I/Fld in which Fd I and Fld are covalently bound by a ruthenium photosensitizer (RuPS) instead of photosystem I. The RuPS is activated by light, whereby an electron is transferred from activated RuPS*: RuPS*-Fd I → FNR and RuPS*-Fld → FNR, respectively. In these cases, an electron transfer occurs within the Fd I/Fld and FNR complexes. In both cases, FNR semiquinone intermediates are observed using electron-spin resonance. In the final step, oxidized NADP^+^ accepts hydride from FADH^−^. This report suggests that photosystem I could sequentially donate one electron to the oxidized Fd I/Fld-oxidized FNR complexes. The electron transfer mechanisms are different from those of Figure 1A and Figure 2A, but this new approach could provide an excellent model system for photosystem I.

## 4. Structure and Properties of Diflavin Reductase Family

**Scheme 2:**
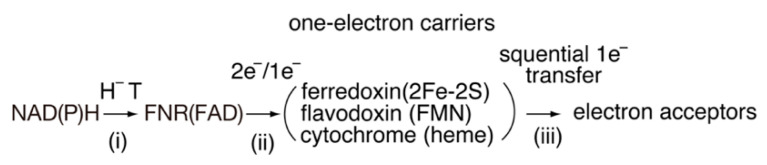
The roles of FNR (FAD) and one-electron carriers in the NAD(P)H-dependent electron transfer systems.

The electron transfer cascade catalyzed by FNR is arranged as presented in Scheme 2. FNR selects a one-electron carrier as a partner of electron transfer systems in which three components, iron sulfur protein ferredoxin, FMN-containing flavodoxin and heme-containing cytochromes, function as a one-electron carrier. Thus, at least three different electron transfer systems might be diversified during the processes of evolution.

The eukaryotic FAD- and FMN-containing diflavin reductase family members are fusion enzymes in which the structure of the FAD/NADPH-binding C-terminal domain is structurally homologous to that of plant FNR, while the FMN-binding N-terminal domain is similar to that of bacterial FMN-containing flavodoxin (Fld). Furthermore, cyt P450 reductase [40,53], methionine synthase (MS) reductase (MS reductase) [54,55], novel reductase 1 (NR1) [56], sulfite reductase (SiR) [57] and P450BM3 [58] are members of the diflavin oxidoreductase family, where the distinct FAD and FMN domains are connected by an additional connecting domain (CD) and flexible hinge (H) [59].

The catalytic cycle of cyt P450 reductase as a prototypic member of the diflavin reductase family is very similar to that of the catalytic cycle of the non-photosystem FNR-Fld system (Figure 3). The catalytic cycle of the membrane-bound type is closely related to that presented in Figure 2B. However, the intermolecular electron transfer between FAD and FMN occurs in the closed form, while its open form donates electrons to cyt P450s in a stepwise manner [12]. On one hand, cyt P450 catalyzes the consumption of one molecule of oxygen/molecule of substrate (RH); one atom of this oxygen molecule inserts into the product (ROH), and the other undergoes two equivalents of reduction: RH + 2e^−^ + O_2_ + 2H^+^ → ROH + H_2_O. On the other hand, methionine synthase (MS) reductase [55] and novel reductase 1 (NR1) [56] are soluble forms that lack the N-terminal anchors domain. For these enzymes, the catalytic cycle is similar to that of the membrane-bound form [14].

In contrast, the reductase domain of NOS isoforms contains additional regulatory elements within the C-terminal reductase domain that control the electron transfer through Ca^2+^-dependent calmodulin (CaM) binding [12,14,60]. The mechanism of FAD reduction by NADPH is closely related to that of mammalian cyt P450 reductase. However, the neuronal (nNOS) and endotherial (eNOS) isoforms are activated by Ca^2+^/CaM binding [60]. On the other hand, the inducible (iNOS) isoform tightly binds Ca^2+^/CaM, and iNOS activity is independent of intracellular Ca^2+^ concentrations. Thus, the iNOS isoform is likely to have functions and mechanism similar to those of cyt 450 reductase [60].

## 5. Catalytic Cycle of Cyt *b*_5_ Reductase

Mammalian NADH-cytochrome *b*_5_ reductase (cyt *b*_5_ reductase) is an FAD-containing flavoprotein that donates electrons to cytochrome *b*_5_ (cyt *b*_5_), and reduced cyt *b*_5_ donates electrons to terminal desaturases [14]. The three-dimensional structure of the protein reveals two distinct FAD and NADH domains [61]. The overall structure is highly conserved with respect to that of bacterial flavodoxin reductase (FldR) [13]. However, the Ser-Glu/Asp pair conserved in the plant FNR family is not involved in the function of this enzyme [13], thus indicating that cyt *b*_5_ reductase is not a functional member of the FNR family, despite the knowledge that this enzyme is a structural member of FNR. Additionally, the N5 atom of the isoalloxazine ring of FAD is stabilized via hydrogen bonding with Tyr65 and Thr66, which leads to the highly conserved His49 (FAD-N5**^…^**Tyr65/Thr66**^…^**His49**^…^**cyt *b*_5_) that is located near the cyt *b*_5_-binding site [61]. Additionally, cyt *b*_5_ reductase-cyt *b*_5_ complexes are stabilized by the formation of salt bridges, whereas a ~30 mV negative shift of cyt *b*_5_ reductase redox potential via the binding of cyt *b*_5_ is observed [62]. However, the reduced and semiquinone states of FAD are stabilized by the binding of NAD^+^ [63], while Gutiérrez-Merino et al. [64] reported that the distance between the iron heme group of cyt *b*_5_ and highly conserved His is 12.4 Å based on a docking model (see Figure 3b of Reference [64]).

**Figure 4:**
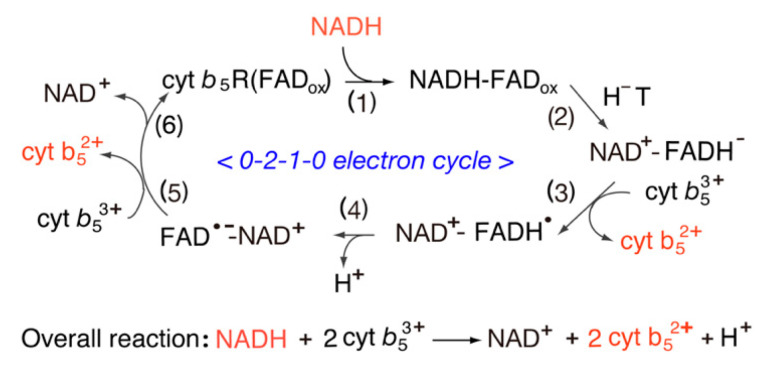
Proposed catalytic cycle of NADH-cyt *b*_5_ reductase. Note that cyt*b*_5_R (FAD_ox_) indicates oxidized cyt *b*_5_ reductase.

The catalytic cycle of cyt *b*_5_ reductase is significantly different from that of FNR (Figure 4). In the first step, FAD accepts a hydride from NADH with the concomitant production of a long-wavelength-absorbing NAD^+^-reduced FAD charge-transfer complex (NAD^+^-FADH^−^), and it is oxidized through semiquinone intermediates by cyt *b*_5_ in two one-electron transfer steps. The neutral semiquinone (FADH^•^) resulting from the first step is then rapidly deprotonated: FADH^•^ → FAD^•−^ + H^+^ (Figure 4) [65]. When the oxidized enzyme is mixed with equimolar NADH plus cyt *b*_5_ in the electron-spin-resonance-equipped stopped-flow apparatus, NAD^+^-FAD^•−^ is observed: cyt *b*_5_ reductase (FAD_ox_) + cyt *b*_5_^3+^ + NADH → cyt *b*_5_ reductase (NAD^+^-FAD^•−^) + cyt *b*_5_^2+^ + H^+^ [63]. On the other hand, Kimura et al. [65] constructed a Thr66Val mutant in which the hydroxyl group of Thr66 was replaced with a methyl group. This mutant enzyme stabilized the neutral semiquinone form FADH^•^, and its activities for ferricyanide and cyt *b*_5_ were approximately 9% and 4% of that of the wild type, respectively. This suggests that the anionic form of FAD semiquinone is more active than the neutral form. However, in addition to the broad band at 500–700 nm, its neutral semiquinone form exhibits an additional peak at ~520 nm, that is also observed in the neutral semiquinones of the FNR [28,45] and NOS isoforms [66,67]. Thus, the Thr66Val mutants may exhibit a reaction mechanism similar to that of FNR (see Figure 1B).

Cyt *b*_5_ reductase, cyt *b*_5_ and cyt P450 reductase are present in higher plants [68,69]. Plant cyt P450 reductase and mammalian cyt P450 reductase can donate an electron to cyt *b*_5_ [53,68], while a novel mammalian 58 kDa flavoheme protein (NADH-cyt *b*_5_ oxidoreductase; Ncb5or) is a fusion enzyme that contains the cyt *b*_5_-like domain at the N-terminus and the cyt *b*_5_ reductase-like domain at the C-terminus [70], which are connected by a hinge region.

The soluble cyt *b*_5_ reductase-cyt *b*_5_ pair donates electrons to hemoglobin and myoglobin [71,72], while the mammalian cyt *b*_5_ reductase-cyt *b*_5_ electron transfer system donates an electron to cytoglobin (Cygb) [73]. The redox potential of Cygb (−37 mV) is more negative than that of hemoglobin (+77 mV) [74], suggesting that an oxygenated Cygb (Fe^2+^-O_2_) is more unstable than hemoglobin. Thus, oxygenated Cytg (Fe^2+^-O_2_ ⇌ Fe^3+^-O_2_^•−^) reacts with the ^•^NO radical, resulting in the nitrate ion (NO_3_^−^) via an intermediate, Fe^3+^-OONO^−^ (Fe^3+^-O_2_^•−^ + ^•^NO → Fe^3+^-OONO^−^). This mammalian nitrate ion formation system provides a pathway to regulate NO concentrations in response to oxygen tension [75].

**Figure 5:**
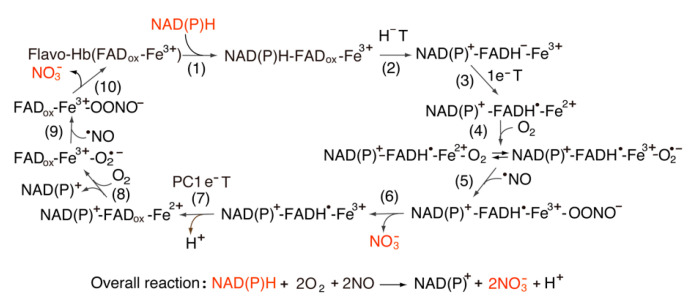
Proposed catalytic cycle for flavohemoglobin.

The bacterial flavohemoglobin (flavoHb) system regulates NO concentrations. The structure exhibits two domains, of which a heme-binding module belongs to the globin family, and an FAD domain shares the FNR-like module. Thus, this enzyme is a fusion enzyme derived from FNR and bacterial hemoglobin [76]. The redox potentials of FAD and heme are ~−150 mV and ~−120 mV, respectively [77]. This value of heme iron is more negative than that of Cygb. As shown in Figure 5, the oxygenated forms are formed in two steps of the catalytic cycle (steps 4 and 8) [78,79]. The equilibrium between Fe^2+^-O_2_  ⇌  Fe^3+^-O_2_^•−^ depends on the right-hand side depending on the negative shift in the redox potentials. It is likely that the Fe^3+^-O_2_^•−^ state reacts with the ^•^NO molecule as described in Cytg. In steps 6 and 10, the nitrate ion, NO_3_^−^, is formed via an intermediate, Fe^3+^-OONO^−^. In addition, the amino acid sequence exhibits the conserved Ser and Glu/Asp pair that is found in FNR, cyt P450 reductase and NOS isoforms.

**Figure 6:**
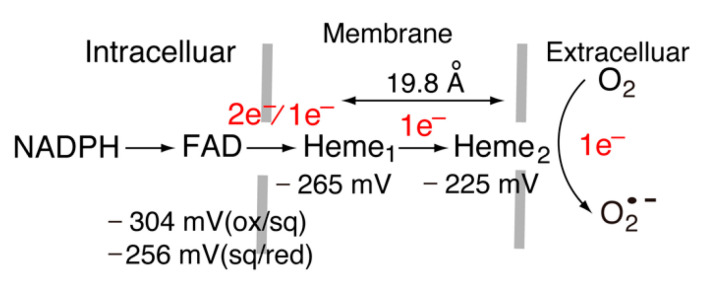
Membrane-bound NADPH-oxidase.

Another interesting example is the membrane-bound NADPH-oxidase (NOX) [80], in which two electrons are transferred from NADPH to molecular oxygen, resulting in a superoxide anion, O_2_^•−^ (Figure 6). The mammalian NOX family includes several isoforms, NOX1-5 and Duoxl 1-2, which are regulated via protein–protein interactions. Recently, Magnani et al. [81] reported the crystal structure of the catalytic FNR-like FAD- and heme-binding domains of *Cylindrospermum stagnale* NOX5, which share six heme-containing transmembrane helical domains and a C-terminal cytosolic FAD-containing dehydrogenase domain (DH) core, as compared with the mammalian NOX family. In addition, the structure supports a linear ET sequence from NADPH to oxygen as shown in Figure 6. The FNR-like FAD domain acts as a converter from the two-electron donor NADPH to one-electron acceptor heme_1_, as the dimethylbenzene edge of FAD can interact with the propionate chains of heme_1_. A distance of 19.8 Å separates the metal centers of the two heme groups of the transmembrane, and the distance between two heme edges is 6.4 Å, indicating electron tunneling, and Trp378 could mediate the electron transfer between two hemes as a radical intermediate (Trp^•−^). A cavity with groups putatively interacting with dioxygen was also proposed (see Figure 6 of Reference [81]), in which oxygen directly accepts an electron from the heme_2_ edge. This model supports the outer-sphere mechanism proposed by Isogai et al. [82].

## 6. Single-Electron Reduction of Quinone Compounds

**Figure 7:**
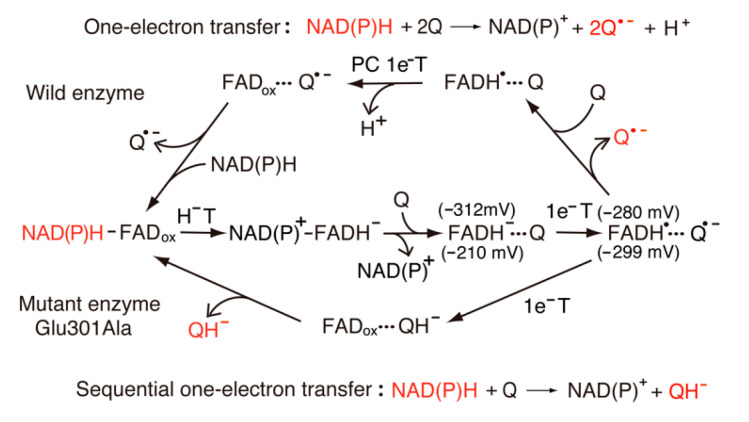
Proposed catalytic cycle of wild and mutant FNR Glu301Ala.

In addition to physiological acceptors, FNR catalyzes the single-electron reduction of exogenous quinone compounds (Figure 7). Iyanagi and Yamazaki [16] demonstrated that plant *L*FNR catalyzes the one-electron reduction of quinones. This reaction is treated as an “outer-sphere electron transfer” model [18]. The conserved Ser80-Glu301 pair that stabilizes the neutral FAD semiquinone presents in the FAD binding site. Thus, the mutation of Glu301Ala causes significant one-electron redox potentials and the destabilization of neutral FAD semiquinone as compared with the wild-type enzyme [31]. It is likely that quinones interact with Tyr303 through hydrogen bonding and accept electrons directly from the isoalloxazine ring of FAD. Čenas and coworkers [19] reported that *Anabaena* PCC7119 FAD-containing FNR catalyzes the one-electron reduction of quinones. This reaction is modulated by the stability of diradical intermediates, such as FADH^•−^···Q^•−^, where the multi-step (e^−^, H^+^, e^−^) model is proposed for the two-electron reduction of quinones. In the wild-type enzymes, the one-electron reduction potentials of FAD are −280 mV for *E*_on/sq_ and −312 mV for *E*_sq/red_, and the value of *E*_on/sq_ is higher than that of *E*_sq/red_ [31]. Thus, a one-electron transfer reaction is a major process.

The rate constants for electron transfer from flavin to a series of quinones derivatives that have different one-electron reduction potentials were studied by Čenas and coworkers [19]. The log *k*_cat_/*K*_m_ vs. one-electron reduction potentials of quinones by FNR exhibit scattered parabolic dependences on *E*_on/sq_ $\left(E_{7}^{1}\right)$, and the data indicate an “outer-sphere” electron transfer model, as expected according to the Marcus theory [83]. In contrast, the redox potentials of mutant enzyme Glu301Ala are −299 mV for *E*_on/sq_ and −210 mV for *E*_sq/red_ [31]. This reverse shift in the one-electron potentials modulates the rate constants for the initial one-electron transfer (FADH^−^ + Q → FADH^•^ + Q^•−^) and the second one-electron transfer (FADH^−^ + Q^•−^ → FAD + QH^−^). The percentage of the one-electron flux in the quinone (Q) reduction by the mutant enzyme is approximately 50%. In the wild enzyme, Q^•−^ is rapidly removed from the FADH^•^···Q^•−^ radical pair, while in the mutant enzyme, quinone reduction includes ~50% of two sequential one-electron reductions, thus resulting in QH^−^, as shown in Figure 7. In addition to *Anabaena*, Ferredoxin: NADP+ oxidoreductase in *Plasmodium falciparum* catalyzes the typical one-electron reduction of quinones [84].

In addition to FNR, enzymes such as cyt P450 reductase (FAD-FMN) [15,17,18,85,86] and the reductase domain (FAD-FMN) of NOS isoforms catalyze the one-electron reduction of quinones [18,67,86,87,88,89,90]. In these enzymes, quinone compounds can mainly accept an electron from a low-potential FAD (Table 1). However, menadione (−200 mV for *E*_on/sq_) can accept an electron from the two flavins in the cyt P450 reductase and inducible NOS reductase domain [86]. Thus, it is likely that quinone compounds can interact with aromatic amino acids, where the isoalloxazine ring of the FMN domain is surrounded by Tyr178 and Tyr140 for rat cyt P450 reductase, and Tyr889 and Phe809 for rat neuronal NOS. Quinone compounds can make a weak hydrogen bond (Tyr-OH···O=Q) with Tyr 178 between Tyr-OH and one of the quinone oxygens (rat cyt P450 reductase) and Tyr 889 (rat neuronal NOS), where the quinone compounds directly accept one electron from the isoalloxazine ring of FMN, resulting in a quinone radical. Thus, FMN can share high activity for quinone compounds as a one-electron acceptor/donor. In the NOS isoforms, the one-electron reduction of quinones is activated via CaM binding [87,88]. In particular, the CaM-bound iNOS reductase domain shares a mechanism similar to that of cyt P450 reductase [86], while cyt *c* accepts electrons from the FMN site of both enzymes, indicating the substrate specificity for protein–protein interactions.

The redox properties of FAD-containing cyt *b*_5_ reductase are significantly different from those of plant FNR. As shown in Figure 4, this enzyme donates one electron to quinones via anionic FAD semiquinone (FADH^−^ → FAD^•−^ → FAD). This enzyme directly donates an electron to quinones including menadione (2-Methyl-1,4-naphthoquinone) [91], but an electron transfer is more effective in the presence of cyt *b*_5_: NADH → cyt *b*_5_ reductase → cyt *b*_5_ → Menadione [92]. In addition, the ascorbate free radical (Asc^•−^) is specifically reduced by this enzyme: Asc^•−^ + e^−^ + H^+^ → HAsc^−^ [15].

## 7. Antioxidant Enzymes: NAD(P)H-Quinone-Oxidoreductase (NQO1)

**Figure 8:**
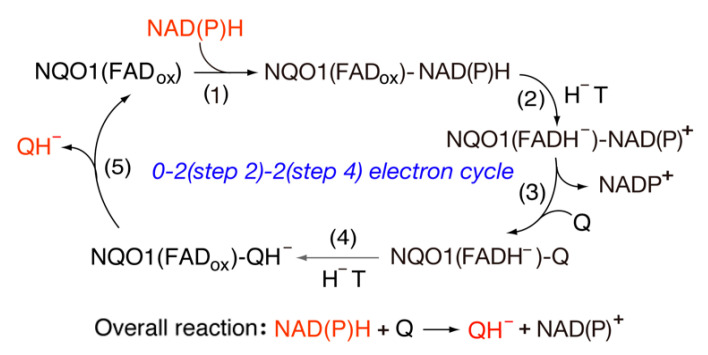
Proposed catalytic cycle of NQO1.

Cytosolic FAD-containing NAD(P)H-quinone oxidoreductase (NQO1), also known as DT-diaphorase, catalyzes the obligatory two-electron reduction of quinones including benzoquinone and naphthoquinone [16,17,18,20,93]. This enzyme also catalyzes the reduction of nitroaromatic compounds [94], but its activity is very low, suggesting that the latter substrates are not fitted in an optimal position to receive a hydride from reduced FAD. The most striking feature of NQO1 is its common flavodoxin-like topology within the catalytic domain (residues 1–220) [95]. Nevertheless, the amino acid sequence similarities are lower, thus indicating convergent evolution at the catalytic site [96]. Quinone derivatives are converted to the hydroquinone form via the direct hydride transfer from the reduced FAD of the active site, where the NAD(P)H and quinone binding share the same site. Thus, the reaction proceeds in two direct hydride transfer steps as a “ping pong” mechanism (Figure 8). The His161 and Tyr155 residues participate in the catalytic cycle, where both residues are arranged in an optimal position to receive a hydride from NAD(P)H to oxidize FAD, and from the reduced FAD to substrate quinones. On the other hand, Mendoza et al. [97] reported that a strong correlation is found between log (*k*_cat_/*K*_m_) and molecular-docking-derived hydride donor–acceptor distance, as expected from the Marcus theory [83]. Recently, Anoz-Carbonell et al. [98] suggested that two active sites in the dimer catalyze two-electron reactions at different rates, indicating the existence of strong allosteric communication between the active sites during the catalytic cycle, while the hydride transfer activities of quinone compounds depend upon the free energy exchange between the FAD/FADH^−^ couple (*E*_m =_ −159 mV) and the Q/QH^−^ (*E*_m_) couple of quinones [99].

Massey and co-workers [100,101] tested a detail mechanism of the two-electron transfer in which they used the ferricyanide [Fe (CN)_6_]^3−^ that functioned as an obligatory one-electron acceptor. The flavin semiquinone intermediate could be observed during the sequential one-electron transfer (FADH^−^ → FAD^•−^ → FAD), where the one-electron reduction potentials of the FAD/FAD^•−^ and the FAD^•−^/FADH^−^ couples were −0.200 V and −0.118 V, respectively. The rate constant of the second step was much faster than that of the first step, thus suggesting that a semiquinone intermediate was not observed. Although the rate constant for ferricyanide is very small, its reactivity with ferricyanide suggests a sequential one-electron transfer mechanism. On the other hand, for the direct hydride transfer, they used a reconstituted 5-deaza-flavin-containing enzyme that could only catalyze two-electron transfers. This enzyme exhibited a specific activity of approximately 5% for quinone reductase activity compared with that of the native enzyme, and it exhibited no activity for ferricyanide. Thus, they confirmed that NQO1 is an obligatory two-electron transfer enzyme (see Figure 8). NQO1 is also present in plants [102] and bacteria [103]. Recently, a new class of quinone reductases (PA1024) from bacterial genes was demonstrated to catalyze a hydride transfer from NAD(P)H to quinone compounds [104]. The overall sequence similarity was only 19% when compared with NQO1, but the overall structure exhibited significant structural similarity to the mammalian NQO1.

The physiological role of NQO1 is not known in detail, but this enzyme could function as an antioxidant for the reactive quinone radical. Ross and Siegel proposed that NQO1 could be modulated by changes in the pyridine nucleotide redox balance, suggesting that NQO1 functions as a redox-dependent molecular switch [105]. In addition, two-electron-reduced quinone compounds are conjugated by phase II drug metabolizing enzymes, including UDP-glucuronosyltransferases (UGTs) and sulfotransferases (SULTs) [21]. UGTs catalyze the glucuronidation of fully reduced quinone compounds, where UDP-glucuronic acid (UDP-GlcUA) is the GlcUA donor: UDP-GlcUA + Q-OH → UDP + Q-O-GlcUA + H^+^. On the other hand, sulfotransferases (SULTs) catalyze the sulfation of fully reduced quinone compounds, where sulfate 3′-phosphoadenosine-5′-phosphosulfate (PAPS) is the sulfate donor: PSPS + Q-OH → 3′-phosphoadenosine-5′-phosphate + Q-O-SO_3_^−^. Thus, NQO1 and UGT/SULT pairs could function as potential antioxidant enzyme systems.

## 8. Evolutionary Aspects of FNR-Containing NAD(P)H-Dependent Enzymes

**Figure 9:**
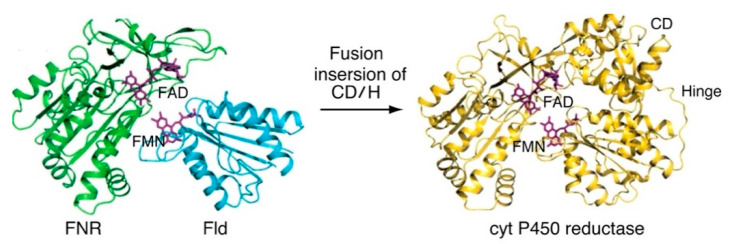
Evolutionary origin of the cyt P450 reductase: (**left**) plant FNR and bacterial Fld redox complex and (**right**) cyt P450 reductase. CD/H indicates the connecting domain (CD) and the hinge (H) [12,14].

**Figure 10:**
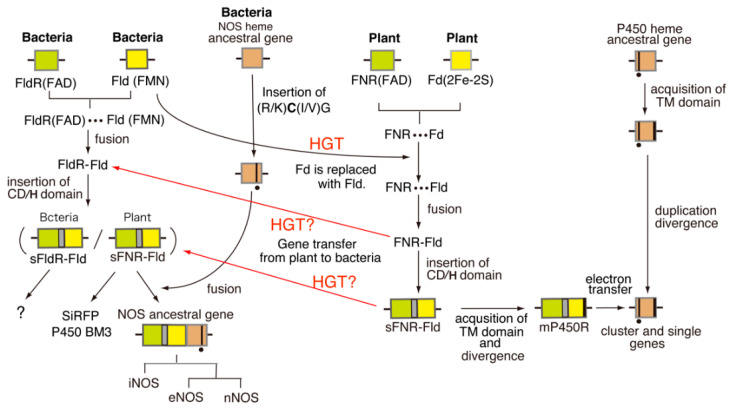
Evolutionary model of the formation of the diflavin reductase family. All domains are arranged in order of the C-terminus (left) to the N-terminus (right), and the FNR (FAD) domain is positioned on the left. FNR-Fd indicates a fusion gene composed of FNR and Fld. sFNR-Fld indicates soluble ancestral FNR-Fld reductase. mP450R, membrane-bound cyt P450 reductase; FldR, flavodoxin reductase; SiFP, bacteria sulfite reductase flavoprotein component; P450BM3, cytochrome P450BM3 from *Bacillus megaterium*. For cyt 450 reductase and NOS isoforms, a dot indicates the conserved proximal cysteine residue, where the heme domains for the bacterial and mammalian NOS isoforms share a sequence motif, (R/K) C (I/V) G in the conserved proximal cysteine residues, which is found in cyt P450 gene family 1 [14]. The CD/H indicates the connecting (C) and hinge (H) domains (gray). TM indicates the membrane-binding domain. The electron transfer occurs from the electron donor NAD(P)H to the final electron acceptor heme iron: NAD(P)H → FAD → FMN/or [2Fe-2S] → Heme iron. HGT (red) indicates the transfer of the bacterial Fld gene to plant cells. HGT? (red) indicates the transfer of the plant FNR-Fld or sFNR-Fld gene to bacterial cells. Thus, the FAD-FMN module of bacterial SiR and P450BM3, mammalian cyt P450 reductase and NOS isoforms are derived from plant FNR-Fld or sFNR-Fld as a precursor gene [14,21].

NAD(P)H-dependent electron transfer systems containing FNR module and electron carrier proteins such as a ferredoxin (Fd) and flavodxin (Fld) are a product derived from the dynamic domain shuffling, mixing and recombination of genes (Scheme 2). The diversity of prokaryote–eukaryote redox enzymes such as cyt P450 reductase and nitric oxide synthase include plant FNR- and bacterial Fld-like domains that have formed through the processes of evolution (see Refs. [12,14] and Figure 9 and Figure 10). In addition to the traditional evolutionary approaches, this review also discusses the perspective of functional approaches.

The bacterial Fld reductase (FldR)-Fld and plant FNR-Fld redox pair systems contributed to the evolution of the diflavin cyt P450 reductase and nitric oxide synthase family (Figure 9 and Figure 10). Therefore, it is a highly interesting aspect that NAD(P)H-dependent electron transfer systems select a one-electron carrier (Scheme 2). The gene transfer between plants and bacteria could create new NAD(P)H-dependent electron transfer systems through evolutionary processes. Fd [2Fe-2S] could be replaced with Fld (FMN) despite their different structure, because Fd and Fld share a common function as a one-electron carrier. It is likely that the bacterial FldR-Fld and plant FNR-Fld redox pairs are the ancestral components of FAD and FMN containing NAD(P)H-dependent ET systems. These systems are arranged as presented in Scheme 2, where at least three NAD(P)H-dependent electron transfer systems might be formed. As an ancestral gene, a gene interaction between plant FNR-Fld and bacterial FldR-Fld redox pairs is a key point in this review, and the possible mechanisms are discussed [12,14,21].

Bacterial FMN-containing Fld is not present in the plant genome [106]. Thus, a plant acquires the Fld gene via horizontal gene transfer (HGT) events from bacteria (see Figure 10). Pierella Karlusich et al. [107] proposed that environmental selection pressures related to iron utilization are involved in the loss of the Fld gene from the plant genome; thus, it is possible that plant cyt P450 reductase derived from the fusion of the plant FNR and bacterial Fld genes (Figure 9 and Figure 10). In the addition to plant and mammalian cyt P450 reductases, the related enzymes are present in bacteria, including sulfite reductase (SiR) [57] and P450BM3 [58]. It is also possible that bacterial diflavin reductases are derived from the gene fusion between bacterial FldR and Fld within bacterial cells. The flavoprotein component (SiR-FP) of bacterial sulfite reductase (SiR) [108,109] and the diflavin module of bacterial P450BM3 [58] are members of the bacterial diflavin reductase family. However, bacterial FldR possesses only 17% identity with plant FNR [13], while the NADPH/FAD domain of bacterial SiR-FP possesses a high sequence identity (~40%) with human cyt P450 reductase [109]. Taken together, plant cyt P450 reductase exhibits a high sequence homology of ~38% amino acid sequence identity with mammalian cyt P450 reductase [110]. These reports suggest that the FAD-FMN modules of SiR-FP and P450BM3 are very closely related to the plant and mammalian diflavin electron transfer systems. Thus, bacterial SiR-FP and the diflavin module of bacterial P450BM3 might have derived from the plant ancestral genes, for which the possibilities of HGT from plants to bacteria are proposed (see Figure 10). It is likely that bacterial NADPH-dependent diflavin modules can be formed, in which bacteria can acquire ancestral plant genes. However, this raises the question of whether the bacterial and plant diflavin domains have independently evolved or not, but it is possible that both gene products function to overlap. On the basis of the amino acid identity, it is most likely that bacterial SiR-FP and the diflavin module of bacterial P450BM3 might have derived from plant ancestral genes, FNR-Fld or sFNR-Fld (Figure 10). The expression of plant ancestral genes in bacterial cells might be more favored than that of bacterial ancestral genes FldR-Fld/sFNR-Fld, suggesting three consecutive interdomain transfer events: *Bacteria* → *Eukarya* → *Bacteria* → *Archaea* [107]. Currently, direct evidence of the gene products derived from bacterial FldR and bacterial Fld has not been reported.

As presented in Figure 9 and Figure 10, the structure and function of FNR and the FAD domain of diflavin reductase are evolutionarily related. The overall structure of FNR is highly similar to that of the FAD domains of cyt P450 reductase [111] and nNOS [112], while the structure of the FMN domain is similar to that of Fld. Taken together, the FAD binding sites of these enzymes share a similar geometry, where the N5 atom of the isoalloxazine ring of FNR(FAD) forms a hydrogen bond with the hydroxyl group of Ser96, which forms hydrogen bonds with Glu302. Based on these structural geometries, Dumit et al. [30] proposed that a proton is transferred from Glu312 via Ser96 to the N5 atom of the deprotonated semiquinone form FAD^•−^ of the flavin, thus making a rapid protonation of the isoalloxazine ring of FAD (FAD^•−^ + H^+^ → FADH^•^) possible, whereby the neutral semiquinone of FAD (FADH^•^) is stabilized. As described above, it is likely that the Glu301Ala mutant affects the redox properties [31]. The FAD ring of cyt P450 reductase shares the Ser457-Asp675 pair [111], and the Ser1176-Asp1393 pair is also conserved in the nNOS isoforms [112]. These Ser-Glu/Asp pairs are also conserved in bacterial FldR [13], bacterial P450BM3 [58] and bacterial SiR-FP [109]. As a common mechanism, these pairs participate in the stability of the neutral semiquinone forms. On the other hand, the N5 atom of the isoalloxazine ring of FMN within the Fld and Fld-like domains forms a hydrogen bond with the peptide carbonyl of conserved Gly residues, where the neutral semiquinone form is stabilized [36,113]. Thus, the one-electron redox potentials of the fully reduced semiquinone couple, *E*_sq/red_, are more negative than the oxidized semiquinone couple, *E*_on/sq_ [36]; the fully reduced semiquinone couple *E*_sq/red_ functions as a one-electron carrier.

In addition to the above discussion, the differences in redox potentials might be reflected in evolution. The values of *E*_on/sq_ and *E*_sq/red_ of FAD of bacterial Fld reductase [114] and bacterial Sir-FP [108] satisfy *E*_sq/red_ > *E*_ox/sq_, while the one-electron redox potentials of plant FNR satisfy *E*_sq/red_ > *E*_ox/sq_, but their values are converted to *E*_ox/sq_ > *E*_sq/red_ in the presence of NADP^+^ (Table 1). In other diflavin reductases, the one-electron redox potentials satisfy *E*_ox/sq_ > *E*_sq/red_, including plant and mammalian cyt P450 reductases [48,115], methionine synthase reductase [115] and NOS isoforms [116], while the one-electron redox potentials of bacterial and eukaryote Fld-like modules satisfy *E*_ox/sq_ > *E*_sq/red_.

Bacterial P450BM3 is a unique fusion enzyme derived from the FAD-FMN reductase domain and cyt P450, where the diflavin reductase is fused to the carboxyl terminus of cyt P450. This reductase domain is structurally analogous to mammalian cyt P450 reductase [117]. The active form of this enzyme is a dimer [118,119]. The FAD-FMN domain of P450BM3 belongs to the cyt P450 reductase family; however, the conserved Gly that forms a hydrogen bond with the N5 atom of the FMN domain is absent [113], and its FMN semiquinone is present in the anionic form, FMN^•−^. The values of the one-electron redox potentials, *E*_ox/seq_ and *E*_sq/red_, of FMN are −240 mV and −160 mV, respectively [120]. Both the fully reduced and semiquinone forms donate an electron to the cyt P450 heme domain. Thus, the catalytic cycle is 0-2-1-0, where the resting state of the enzyme is the fully oxidized form [121]. However, the addition of Gly in the absent position causes a conversion of the one-electron redox potential, and the values of the *E*_ox/seq_ and *E*_sq/red_ of FMN are −198 mV and −245 mV, respectively [122]. This behavior enables two sequential one-electron transfers from both the fully reduced FMN and anionic FMN forms to substrate-bound cyt P450 [14], while the FMN semiquinone of *archaea* Fld is in an anionic form. Prakash et al. [123] suggested that *archaea* Fld is capable of replacing Fd and possesses the potential advantage of sparing iron for abundant iron–sulfur proteins that are essential for growth, thus allowing greater resilience to oxidative damage to be obtained. Taken together, this report leads to the new question of why *archaea* more advanced than bacteria select the anionic FMN semiquinone. In the case of P450BM3, the anionic FMN semiquinone is needed to gain high energy [21], while mammalian cyt P450 reductase and NOS isoforms select the neutral FMN semiquinone, which is not necessarily the case. Taken together, these observations suggest that the electron transfer rate is regulated by the FMN semiquinone states.

The next question regards how P450BM3 has acquired the reductase domain, where this gene might have derived from a fusion domain between FldR and Fld in bacteria. However, the amino acid sequence homologies clearly reveal that the FAD and FMN proteins are closely related to the FAD-FMN domain of plant and mammalian cyt P450 reductases [109,110,117], while the bacterial cyt P450 electron transfer system selects a non-heme iron as a one-electron carrier: NADH → (FAD) → putidaredoxin [2Fe-2S] → cyt P450cam [124,125]. The bacterial cyt P450cin electron transfer system is a three-component system: NADPH → cindoxin reductase (FAD) → cindoxin (FMN) → cyt P450cin [126,127]; this system selects a flavodoxin-like FMN protein as a one-electron carrier. The cindoxin reductase displays strong similarity to NADPH-dependent ferredoxin reductases, and cindoxin reductase is replaced by *Escherichia coli* flavodoxin reductase. Klenk et al. [128] reported two novel cyt P450s in *Arthrobacter sp.*, and they proposed the potential cluster in the genome including two cyt P450s and electron partners flavodoxin and flavodoxin reductase. Thus, the gene organization of bacterial P450BM3 might be a special case. As mentioned above, the cyt P450 reductase-like domain of P450BM3 might have derived from plants via HGT (see Figure 10), although the detailed evolutionary background remains unknown.

In addition to the above discussion, mammalian cyt P450-containing electron transfer systems are divided into two types. The mitochondrial system has three components: NADPH → adrenodoxin reductase (FAD) → adrenodoxin [2Fe-2S] → cyt P450, while the microsomal system has two components: NADPH → cyt P450 reductase (FAD-FMN) → cyt P450. FAD-containing adrenodoxin reductase belongs to the glutathione reductase family, while the FAD module of cyt P450 reductase belongs to the FNR family. The mitochondrial system might have derived from bacteria via endosymbiosis, while the microsomal electron transfer system derived from a fusion between FNR (FAD) and flavodoxin (FMN) [14,21]. However, both electron transfer systems have also evolved synergistically for the biosynthesis of endobiotics, such as steroid hormones and vitamin D [21].

Recently, Zhang et al. [129] reported a new type cyt P450_TT_ electron transfer system in *T. thermophilus* that consists of three domains in the N-to-C-terminal order: N-cytP450-FMN domain-ferredoxin [2Fe2S]-C. The structures of the FMN domain and ferredoxin bear structures similar to those of plant FNR/phthalate dioxygenase reductase and plant Fd, respectively. The electron transfer is NAD(P)H → FMN → ferredoxin [2Fe-2S] → cyt P450_TT_ [130]. However, Yamamoto et al. [131] reported a new cyt P450 electron transfer system that functions in the initial hydroxylation of the cyclohexanecarboxylate degradation pathway. This unique single electron transfer system is N-terminal ferredoxin-[2Fe-2S] domain- FAD-containing FAD domain-FCD like domain-C-terminal cyt P450 domain and is involved in *Paraburkholderia terrae* strain KU-64. They suggested that the electron transfer components of ferredoxin and the FAD domain are similar to those of bacterial biphenyl dioxygenase.

**Figure 11:**
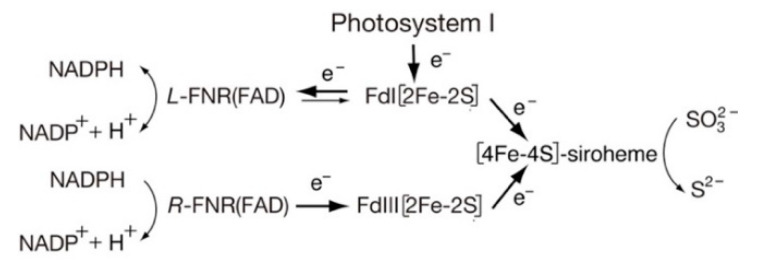
Electron transfer cascades for plant sulfite reductase. Figure was adapted with permission from Refs. [132,133] with some modifications. Copyright year 2005, Hase et al. (2005).

Additionally, it is interesting that sulfite reductase (SiR) systems are present in both the bacteria and plants [132,133]. The reductase domain (SiRFP) of SiR involves a cyt P450 reductase-like diflavin FAD-FMN module that donates electrons to the [4Fe-4S]-heme center in bacteria, while root-type Fd III [2Fe-2S] donates electrons to the [4Fe-4S] heme center in higher plants. Thus, tissue-specific electron transfer systems are divided in bacterial and plant types: Bacterial type, NADPH → SiRFP (FAD-FMN) → SiRHP ([4Fe-4S]-siroheme cluster); plant type, NADPH → *R*FNR(FAD) → Fd III [2Fe-2S] → [4Fe-4S]-siroheme cluster (Figure 11).

Both the electron transfer systems catalyze the six-electron reduction of sulfite (SO_3_^2−^) to sulfide (S^2−^): 3NADPH + SO_3_^2−^ + 3H^+^ → 3NADP^+^ + S^2−^ + 3H_2_O. In addition to the plant-type ET system, a plant utilizes another type of ET system: Photosystem I → Fd I [2Fe-2S] → 4[Fe-4S]-heme center ([4Fe-4S]-HP). Despite the presence of cyt P450 reductase as a diflavin reductase in plants, the FNR(FAD)-Fd III redox pair donates electrons to the [4Fe-4S]-siroheme center. Thus, the presence of different electron transfer systems suggests that these electron transfer systems might have evolved independently. Recently, Tavolieri et al. [109] reported a 30-amino acid hinge/loop in *E. coli* SiRFP, which is significantly longer than the 12-amino acid linker/hinge in cyt P450 reductase [12], thus making more flexibility of the FMN domain possible. Based on the crystal structure and small neutron scattering data [134], they proposed a large rotational and translational motion of the FMN domain, for which they proposed two electron transfer models. A single-electron transfer model is cyt P450-like, NADPH → FAD → cis FMN → SiRHP (*cis* electron transfer to SiRHP), while the second model is unique, in that it shows the intermolecular electron transfer from the FAD domain of one monomer to the FMN domain of the other monomer: NADPH → FAD → trans FMN → SiRHP (*trans* electron transfer to SiRHP). Despite the presence of cyt P450 reductase in higher plants, diflavin-containing SiR does not function in higher plants. It is unknown why plants select the plant-type electron transfer system (Figure 11). However, it is likely that the plant photosystem I-linked path might be more effective than the bacterial type, in which low-potential FdI (−401 mV) can donate electrons to *L*-FNR(FAD) and the [4Fe-4S]-siroheme center.

A cyt P450-like NOS oxygenase is present in bacteria, and this electron transfer system is composed of three redox components: NADPH → flavodoxin reductase (FAD) → flavodoxin (FMN) → cyt P450-like oxygenase [135,136]. However, the NOS proteins are not present in plants. Thus, the diflavin reductase domain of mammalian NOS isoforms might have derived from the bacterial Fld reductase and bacterial Fld (see Figure 10), where the amino acid sequence and structure of the FAD domain are highly similar to those of the mammalian NOS isoforms [112]. The NOS isoforms involve the cyt P450 reductase-like and the cyt P450-like oxygenase domains, and both domains are connected by a calmodulin (CaM)-binding linker region [12]. Additionally, three NOS isoforms, iNOS, nNOS and eNOS, acquired several regulatory elements and co-factor binding sites, and the dimer is their active form [12]. The overall rate-limiting step for NOS isoforms is between the reductase domain and the heme center, and CaM stimulates this step [14]. In addition to these reports, an increase in flavin domain activity causes increases in NO synthase activity [137]. Wang et al. [138] recently demonstrated that the FNR module of NOS isoforms plays a critical role in controlling the electron transfer capacity of the FMN module. As described in P450 BM3, it is likely that the diflavin reductase domain of the NOS isoforms might have derived from plant ancestral genes FNR-Fld or sFNR-Fld via HGT (Figure 10). It remains unknown as to why the fusion proteins derived from bacterial flavodoxin reductase and flavodoxin do not contribute to the formation of the diflavin module.

In plants, the number of cyt P450 reductases includes one to three isoforms within plant species; however, two cyt P450 reductases are predominantly expressed in plant species. Mizutani and Ohta [139] isolated two isoforms from higher plants that exhibited amino acid sequences and redox properties highly similar to those of mammalian cyt P450 reductase. These enzymes can donate electrons to several hundred of plant cyt P450s [140]. In contrast, only one gene is expressed in mammals, and it donates electrons to ~50 microsomal cyt P450s [14,21]. Thus, mutations in cyt P450 reductase influence the function of ~50 microsomal cyt P450s [141]. In addition to cyt P450s, cyt P450 reductase donates an electron to heme-oxygenase [142,143], cyt *b*_5_ [40] and cyt *c* [40], thus indicating diverse functionalities of the electron acceptors [144].

The NOS-type electron transfer system is not present in land plants. Alternatively, NO is produced by nitrate reductase in plants. This enzyme is a single peptide that arises from the gene fusion of cyt *b*_5_ reductase, cyt *b*_5_ and molybdopterin [145]. Nitrate is converted via the two-electron reduction of nitrate to nitrite (NO_3_^−^ + 2e^−^ → NO_2_^−^ + H_2_O), thus resulting in the conversion of nitrite of nitrite to NO via one-electron reduction (NO_2_^−^ + e^−^ → NO + H_2_O). Conversely, bacterial nitrate assimilation includes Fd- or Fld-dependent nitrate reduction systems: Fd or Fld → molybdopterin [146]. Tan et al. [147] recently reported an NADPH-dependent nitrate assimilation system that consists of a single protein. This enzyme includes the diflavin reductase module that donates electrons to molybdopterin: NADPH → (FAD-FMN) → MGD-Molybdopterin-[4Fe-4S]; this system includes the conserved Ser381-Glu614 pair (see Figure S4 of Ref. [147]). The expression of this gene is mainly limited to *Actinobacteria* and *Proteobacteria*, suggesting that this gene has evolved more recently.

## 9. Conclusions

Finally, the gene fusions between plant FNR (FAD) and bacterial Fld (FMN) have adapted to diverse NAD(P)H-dependent electron transfer systems (Figure 9 and Figure 10), among which HGT is the driving force in the evolution of eukaryotic genomes [148]. Although gene transfer from plants to microorganisms is a rare event [149,150], it is one of the possible mechanisms. Both FNR and FDA/Fld have traveled to humans from bacteria over long periods of time [151], and their functions have been conserved. In particular, the cyt P450 reductase-cyt P450 pair system and more organized NOS isoforms including the CaM-binding site are the most excellent natural products [12,14]. Taken together, Figure 9 and Figure 10 shed new light on the origin of the diflavin reductase family.

## Figures and Tables

**Table 1:** Midpoint reduction potentials of FAD and FMN domains.

Enzyme Specicies	*E*_ox/sq_ (mV)	*E*_sq/red_ (mV)	pH
FNR (FAD)			
Spinach (FAD)	−350	−335	7
	−402	−358	8
Spinach (FAD)	−306	−386	8
(in the presence of NADP^+^)			
*Anabaena* (FAD)	−385	−371	8
*Anabaena* Flavodoxin (FMN)	−212	−436	7
Cyt P450 reductase (FAD-FMN)			
Rabbit (FAD)	−290	−365	7
Rabbit (FMN)	−110	−270	7
Human (FAD)	−283	−382	7
Human (FMN)	−56	−274	7
nNOS reductase domain (FAD-FMN)			
Rat (FAD)	−260	−280	7.6
Rat (FMN)	−50	−276	7.6
iNOSreductase domain (FAD-FMN)			
Human (FAD)	−240	−270	7
Human (FMN)	−105	−245	7

**Table 2:** Kinetic parameter of *Pea* and *Anabaena* FNR in NADPH-dependent cytochrome *c* reductase activity.

FNR Species	$K_{cat}^{\mathrm{Fd}}\;(s^{-1})$	$K_{m}^{\mathrm{Fd}}$ (µM)	$K_{cat}^{\mathrm{Fd}}/K_{m}^{\mathrm{Fd}}$ (µM s^−1^)	$K_{cat}^{\mathrm{Fld}}\;(s^{-1})$	$K_{m}^{\mathrm{Fld}}$ (µM)	$K_{cat}^{\mathrm{Fld}}/K_{m}^{\mathrm{Fld}}$ (µM s^−1^)
*Pea* FNR	139	6.5	21.3	30.6	16.7	1.8
*Anabaena* FNR	200	11	18.2	23.3	33	0.7
